# Small molecule targeting FOXM1 DNA binding domain exhibits anti-tumor activity in ovarian cancer

**DOI:** 10.1038/s41420-022-01070-w

**Published:** 2022-06-09

**Authors:** Zaixin Zhang, Si-tu Xue, Yan Gao, Yingwei Li, Ziying Zhou, Jing Wang, Zhuorong Li, Zhaojian Liu

**Affiliations:** 1 Key Laboratory of Experimental Teratology, Ministry of Education, Department of Cell Biology, School of Basic Medical Sciences, Cheeloo College of Medicine, Shandong University, Jinan, China; 2 Institute of Medicinal Biotechnology, Chinese Academy of Medical Sciences & Peking Union Medical College, Beijing, 100050 China; 3 Department of Obstetrics and Gynecology, Qilu Hospital, Cheeloo College of Medicine, Shandong University, Jinan, 250012 Shandong Province China

**Keywords:** Ovarian cancer, Small molecules

## Abstract

FOXM1 is a potent oncogenic transcription factor essential for cancer initiation, progression, and drug resistance. FOXM1 regulatory network is a major predictor of adverse outcomes in various human cancers. Inhibition of FOXM1 transcription factor function is a potential strategy in cancer treatment. In this study, we performed structure-based in silico screening to discover small molecules targeting the FOXM1 DNA-binding domain (DBD). Compound XST-20 was identified to effectively suppress FOXM1 transcriptional activities and inhibit ovarian cancer cell proliferation. XST-20 directly interacts with the FOXM1 DNA-binding domain determined by SPR assay. Furthermore, XST-20 was found to significantly reduce the colony-forming efficiency and induce cell cycle arrest and apoptosis. Our study provides a lead compound of FOXM1 inhibitor which may serve as a potential targeted therapy agent for ovarian cancer.

## Introduction

Ovarian cancer has the highest mortality of all gynecologic cancer, with 239,000 new cases and 152,000 deaths reported annually [1]. Despite the improvements in surgery and chemotherapy in recent years, more than 80% of patients with advanced-stage ovarian cancer relapse within 18–24 months [2]. Poly ADP-ribose polymerase inhibitors (PARPi) have been shown effective in ovarian cancer patients with homologous recombination deficiencies (HRD). Nevertheless, patients who received PARPi therapy will eventually develop resistance to the treatment [3]. The mechanism underlying PARPi resistance in ovarian cancer remains to be elucidated.

FOXM1 is an oncogenic transcription factor which is elevated in several types of cancers, while its expression level in normal tissues is low [4]. FOXM1 regulatory network predicts adverse outcomes in 18,000 cases of 39 human malignancies [5]. FOXM1 regulates the expression of several targets involved in tumor growth, angiogenesis, and invasion. FOXM1 promotes proliferation in liver cancer cells through transcriptional upregulation of CCNB1 [6]. FOXM1 stimulates glioblastomas angiogenesis upon activation of VEGF [6]. FOXM1 modulates epithelial-mesenchymal transition by targeting Snai1 in liver cancer [7]. FOXM1 is commonly elevated and its high level predicts a worse prognosis for ovarian cancer [8]. FOXM1 enhances aggressiveness via transcriptional upregulation of DLX1 and EXO1 in ovarian cancer [9, 10]. Our group report that Cyclin F and KIF20A are transcriptionally upregulated by FOXM1 in ovarian cancer [11].

The Discovery of small-molecule compounds selectively targeting the DNA binding domain (DBD) makes transcription factors druggable. A small-molecule compound targeting DBD of STAT3 has been shown anti-tumor effect [12]. A compound that directly targets HSF1 (heat shock transcription factor 1) inhibits its transcriptional activity, which induces cell death [13]. Small molecules that physically interact with the DBD of FOXO3 block its transcriptional program in human cells [14]. FDI-6 is a small-molecule compound that targets FOXM1–DNA interaction and exhibits an anti-tumor effect [15]. A DNA aptamer targeting FOXM1 DBD suppresses its transcriptional activity and inhibits cancer cell proliferation [16]. Nevertheless, there is still no FOXM1 inhibitor available for clinical use.

Here, we performed structure-based in silico screening to discover small molecules targeting FOXM1 DBD. The target used in this study was the 3D structure of FOXM1. 277324 structures from the SPECS compound library were screened in silico against the FOXM1 using the LibDock module of Discovery Studio 2.5(DS). We selected 15 candidates for bioassay based on molecular docking and biological evaluation. XST-20 was identified to effectively suppress FOXM1 transcriptional activities and exhibit anti-tumor activity.

## Results

### Conserved residues analysis of FOXM1 DBD

**Fig. 1:**
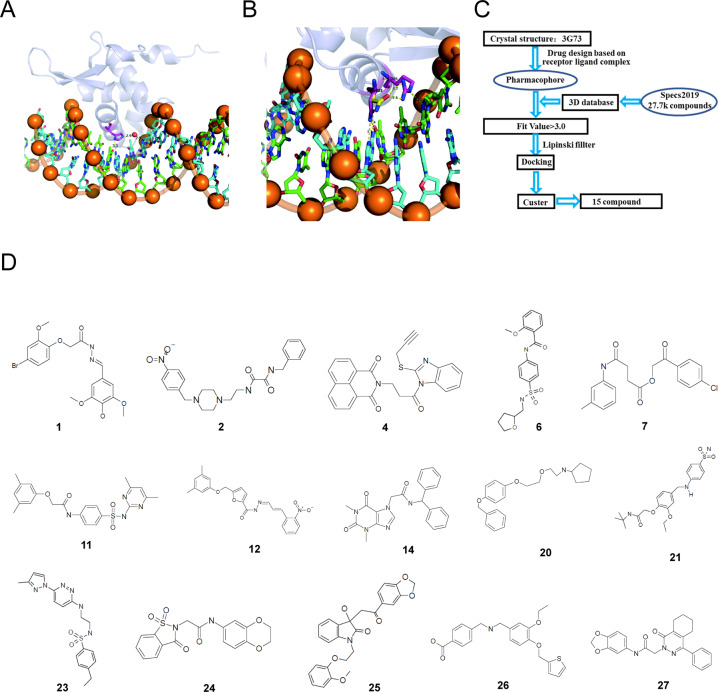
Conserved residues analysis of FOXM1 DNA binding domain and screen small molecules selectively targeting FOXM1 DBD. **A** Shows the hydrogen bonding interactions of H287 with the DNA bases. The sidechain of H287 interacted with the DNA base via a direct H-bond and an indirect H-bond mediated by a water molecule. **B** Shows the interactions of N283 and R286 with DNA. The sidechain of N283 adopted two different conformations. With the vertical conformation (purple), the acid amide group of N283 formed two H-bonds with DNA. In contrast, with the horizontal conformation (yellow), N283 made little interaction with DNA but two H-bonds with the guanidine group of R286. As for R286, it contributed one H-bond with DNA. **C** The analysis steps were performed to select the final 15 candidates from the space compound library. **D** Chemical structures of the 15 candidates.

FOXM1 is a transcriptional factor that binds to DNA consensus sequences through a highly conserved DBD. Asn-283, Arg-286, and His-287 are conserved residues that contribute to protein-DNA binding [17]. To better understand the interaction between these residues and DNA, the PyMOL Molecular Graphics System was employed. The sidechain of His-287 interacted with the DNA base via a direct H-bond and an indirect H-bond mediated by a water molecule (Fig. 1A). Presumably, the mutation of H287 may disrupt the H-bonding network, thereby diminishing the binding affinity of FOXM1 to DNA. In addition, the sidechain of Asn-283 adopted two different conformations. With the vertical conformation (purple), the acid amide group of Asn-283 formed two H-bonds with DNA. In contrast, with the horizontal conformation (yellow), Asn-283 made little interaction with DNA but two H-bonds with the guanidine group of Arg-286. As for Arg-286, it contributed one H-bond with DNA (Fig. 1B). It can be inferred that the mutation of Asn-283 decreases the binding affinity of FOXM1 with DNA. Although the mutation of Arg-286 abolishes its H-bonding interaction with DNA, this may increase the interaction of Asn-283 with DNA by improving the possibility that Asn-283 adopts the vertical conformation. The analysis confirmed the critical role of Asn-283, Arg-286, and His-287 in FOXM1–DNA interaction.

### High-throughput screen small molecules selectively targeting FOXM1 DBD

In order to discover new molecules that block FOXM1 transcriptional activity through binding its DBD, we used the LibDock module of Discovery Studio 2.5(DS) to screen 277324 compounds. The 3D structure of FOXM1was retrieved from the Protein Data Bank (PDB: 3G73). The molecules from the SPECS database were filtered based on Lipinski and Veber rules, and 17,876 structures were excluded. A total number of 38,737 compounds were successfully docked, and we selected 400 top hits for further evaluation using the DS 2.5 software package [18]. Of these 400 molecules, 68 molecules were selected for further verification based on biomolecular interaction and commercial availability. A diagram of structure-based in silico screening to discover small molecules targeting the FOXM1 DNA binding domain was shown in Fig. 1C. Finally, 15 candidates (Fig. 1D and Table S1) were obtained for further investigation.

### Validation of small molecular compounds that inhibit FOXM1

**Fig. 2:**
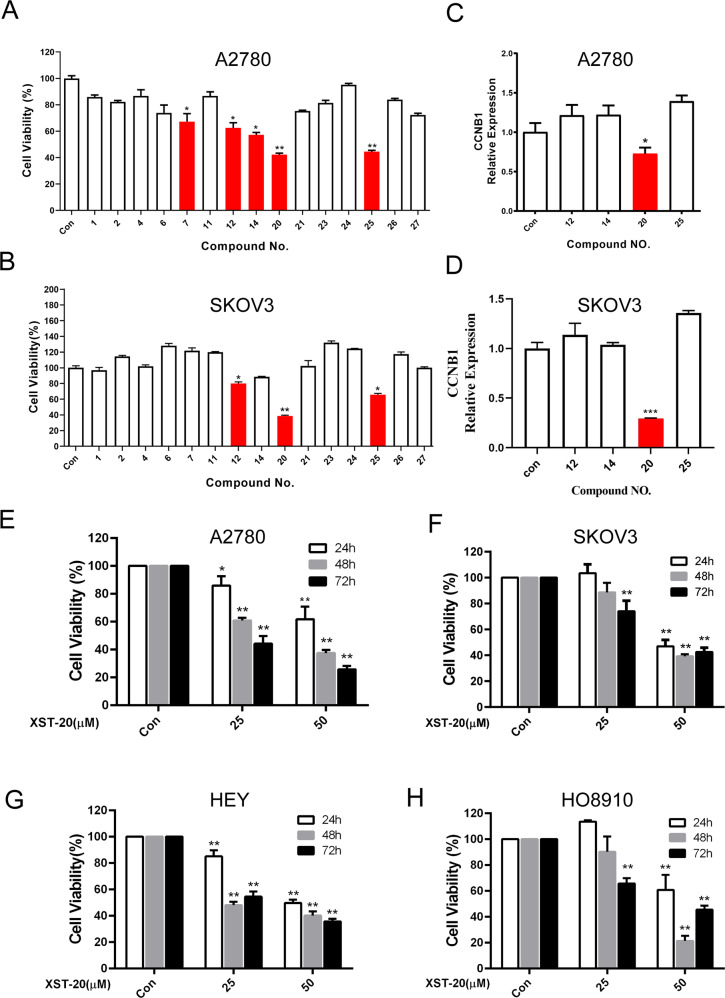
Validation of small molecular compounds that inhibit FOXM1. **A**, **B** The inhibitor effect of 15 candidate compounds on ovarian cancer cell lines were measured by MTT assay. **C**, **D** Expression level of CCNB1 was detected by qPCR in A2780 cells treated with compounds. **E**–**H** Inhibitor effect of XST-20 on four ovarian cancer cell lines was evaluated by MTT assay. Data were presented as means ± S.D. *p < 0.05, **p < 0.01, ***p < 0.001, and ****p < 0.0001.

We first evaluated the inhibitor effect of 15 compounds on ovarian cancer cell lines by MTT assay. As shown in Fig. 2A, B, hit compounds XST-12, XST-20, and XST-25 (40 μm; 48 h) exhibited a significant inhibitor effect on both A2780 and SKOV3 cells. We next measured known FOXM1 downstream targets CCNB1 and CDC25B expression by qPCR in ovarian cancer cells treated with compounds (40 μm; 48 h). Compound XST-20 was found to suppress both CCNB1(Fig. 2C, D) and CDC25B (Fig. S1A, B) expression levels, indicating XST-20 inhibits FOXM1 transcriptional activity. The inhibitor effect of XST-20 on ovarian cancer cells was further validated by MTT assay. As shown in Fig. 2E, XST-20 inhibited proliferation of ovarian cancer cells in a dose and time-dependent manner. The results suggest that XST-20 might be a selective inhibitor of FOXM1 in ovarian cancer.

### Potential binding mode of XST-20 with FOXM1 DBD

**Fig. 3:**
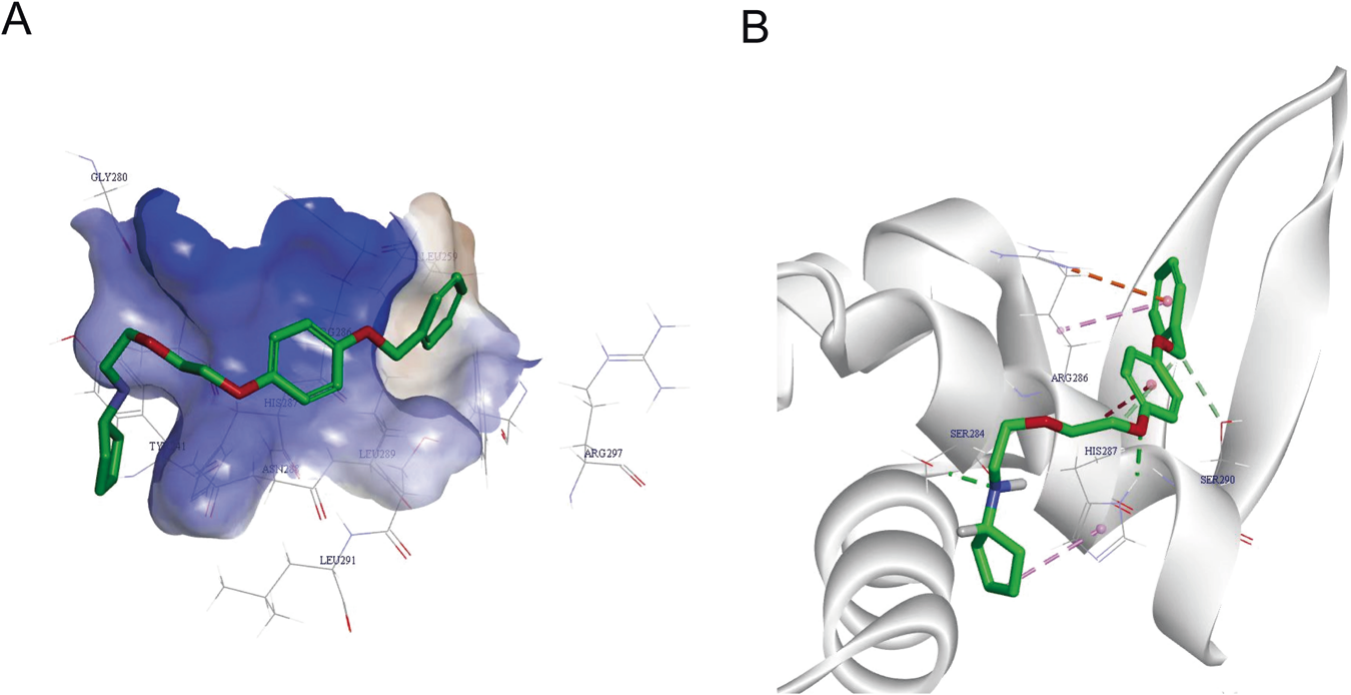
Potential binding mode of XST-20 with FOXM1 DNA binding domain. **A** The orientation of XST-20 in the active site of FoxM1. The co-crystal ligand is represented as stick diagrams with carbon atoms in green. Amino acid residues surrounding the active site are shown inline. **B** The binding mode of XST-20 in the active site of FoxM1. The ligand is represented as stick diagrams with carbon atoms in green. Relevant amino acid residues in the binding site are shown inline. Green dashed lines represent conventional hydrogen bond interactions. Pale green dashed lines represent carbon–hydrogen bond interactions. Pink dashed lines represent Pi–alkyl interactions. Red dashed lines represent Pi–cation interactions.

To further verify whether XST-20 interacted directly with FOXM1 DBD. CDOCKER module in DS4.0 was employed for molecular docking. Importantly XST-20 was integrated with the FOXM1 DBD, XST-20 interacted with the amino acid residues of His-287, Arg-286, and Ser290 (Fig. 3A, B). Among them, the oxygen ion in the diethyl ether chain had a hydrogen bond interaction with His-287, the benzyloxyanisole group had a weaker Pi-ion interaction with the amino acid residue, and the cyclopentylamino group did not interact with the protein.

### XST-20 specifically blocks FOXM1 DNA-binding and inhibits its transcriptional activity

**Fig. 4:**
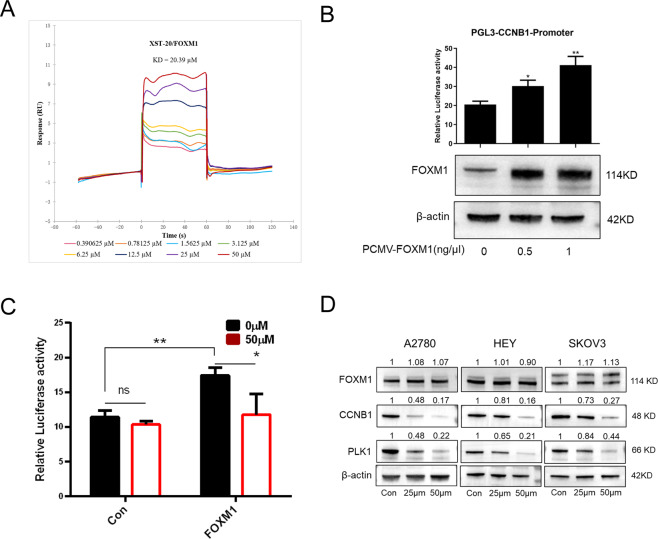
XST-20 specifically blocks FOXM1 DNA-binding and inhibits its transcriptional activity. **A** Interaction between XST-20 and FOXM1 (222–360) peptide was detected by surface plasmon resonance (SPR) assay. **B** Luciferase activity of CCNB1 promoter was evaluated in cells with FOXM1 overexpression. **C** Luciferase activity of CCNB1 promoter was evaluated in cells with FOXM1 overexpression and control group treated with XST-20. **D** Expression levels of FOXM1 and downstream targets were Measured by western blot in three ovarian cancer cell lines (A2780, HEY, and SKOV3) treated with XST-20. Data were presented as means ± S.D. *p < 0.05, **p < 0.01.

In order to verify the specificity of XST-20 on FOXM1 DBD, a surface plasmon resonance (SPR) assay was used to analyze the binding affinity between XST-20 and FOXM1 protein. As a result, the binding of FOXM1 (222–360) peptide with XST-20 exhibited time-dependent saturation, and the *K*_D_ value was ~20.39 μM (Fig. 4A). We next examined whether XST-20 could reduce the transcriptional activity of FOXM1. To this end, we constructed pGL3 CCNB1(a well-known FOXM1 target gene) reporter and a luciferase assay was performed. As expected, overexpression of FOXM1 significantly enhanced luciferase activity of the CCNB1 promoter constructs (Fig. 4B) and the enhanced luciferase activity induced by FOXM1 was decreased upon XST-20 treatment (Fig. 4C). Furthermore, we performed an immunoblot to measure the expression level of FOXM1 and its downstream targets treated with XST-20. As shown in Fig. 4D, XST-20 (25 and 50 μM) significantly reduced the protein level of CCNB1 and PLK1 while FOXM1 protein level was not changed in three ovarian cancer cell lines. These findings provide strong evidence that XST-20 directly interacts with the FOXM1 DNA-binding domain and block its transcriptional activity.

### XST-20 exhibits anti-tumor effect in ovarian cancer cells

**Fig. 5:**
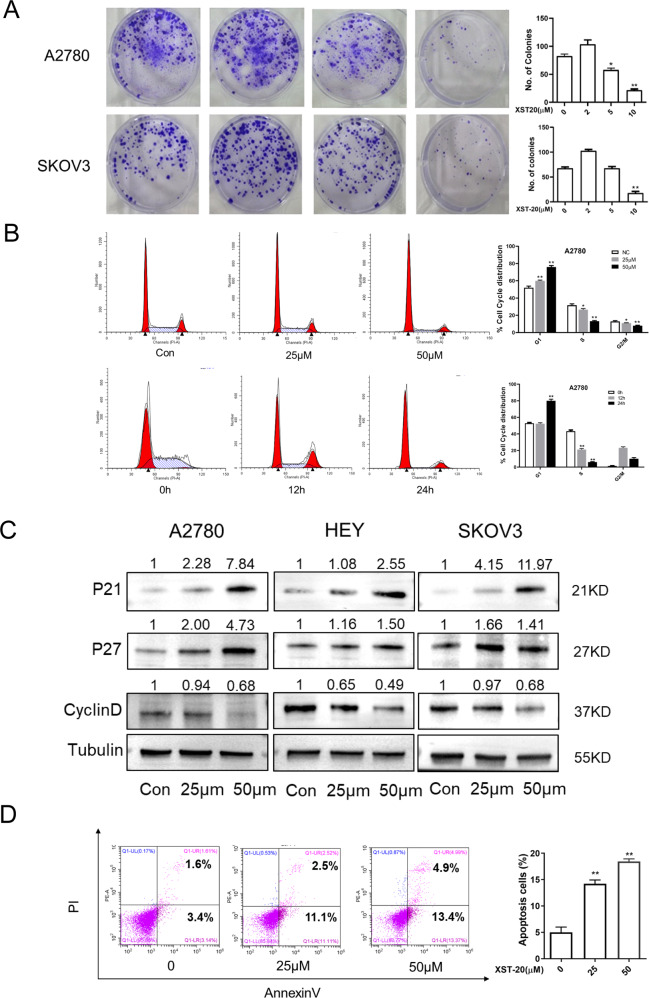
XST-20 exhibits an anti-tumor effect in ovarian cancer cells. **A** Clonogenic assay were conducted to measure the inhibitor effect of XST-20 on ovarian cancer cells. **B** Cell cycle distribution was examined by flow cytometry in A2780 cells treated with XST-20. **C** Proteins that are involved in the regulation of G1/S progression were analyzed by western blot in A2780, HEY, and SKOV3 cells treated with XST-20. **D** A2780 cells were treated with XST-20 and apoptosis was measured by flow cytometry with annexin V staining. Data were presented as means ± S.D. *p < 0.05, **p < 0.01 compared with the control group.

To assess the anti-tumor effect of XST-20 in ovarian cancer cells. We then performed a clonogenic assay on ovarian cancer cells with various concentrations of XST-20 treatment. As shown in Fig. 5A, XST-20 dramatically decreased the number and size of colonies in A2780 (5 μM) and SKOV3 (10 μM) cells. Cell cycle phase distribution was then evaluated by using flow cytometric DNA content analysis in A2780 cells. As expected, the percentage of cells in S and G2 was reduced, whereas the percentage of cells in G1was increased in cells following treatment with XST-20 (Fig. 5B). Further, cell cycle-related proteins were measured by immunoblot in ovarian cancer cells treated with or without XST-20. We found the protein level of cyclin D was downregulated accompanied by upregulation of p21 and p27 in A2780 and SKOV3 cells treated with XST-20 (Fig. 5C). To address whether XST-20 exerts anticancer activities by inducing apoptosis against ovarian cancer cells. Apoptosis was analyzed by flow cytometry through annexin V/PI staining in A2780 cells treated with or without XST-20. As shown in Fig. 5D, the percentage of apoptotic cells was increased upon XST-20 treatment. These results together demonstrated that XST-20 exerts its anticancer activities by inducing cell cycle arrest and apoptosis in ovarian cancer cells.

## Discussion

As a well-known oncogenic transcriptional factor, FOXM1 is involved in tumorigenesis and progression in cancers. Silencing of FOXM1 inhibits cancer cell proliferation and induces apoptosis [19]. In this study, we performed a high-throughput in silico docking screen to select compounds that target the FOXM1 DNA binding domain. We identified XST-20 as a leading compound which could specifically block FOXM1 DNA-binding and inhibits its transcriptional activity. Importantly, XST-20 effectively inhibits ovarian proliferation and induces apoptosis of cancer cells in vitro.

FOXM1 signaling is a major predictor of adverse outcomes in many human malignancies. Originally, FOXM1 was identified as a proliferation-associated transcription factor, but more and more evidence reveals that FOXM1 is also involved in angiogenesis, invasion, stemness, and chemoresistance. FOXM1 promotes tumor initiation [20], EMT [21], and angiogenesis [6]. FOXM1 promotes chemoresistance through transcriptional upregulates UHRF1 expression [22]. FOXM1 recruits AURKA as a transcriptional factor that promotes breast cancer stem cell self-renewal and drug resistance [23].

Transcription factors are potential drug targets for cancer therapy. However, they are traditionally been considered too difficult to target. Mechanistic studies of the transcription machinery and technological advances improve transcription factor druggability. Small-molecule in S3-54A18 binds directly to the DBD and inhibits the DNA-binding activity of STAT3 [24]. Small-molecule inhibitors of SOX18 are also developed by disrupting the DNA binding domain [25]. Our group identified a small-molecule inhibitor targeting MDM2–p53 interaction [26]. Santonin-related compound 2 blocks p65 nuclear translocation of via targeting cysteine 38 [27]. FDI-6 is selected by a fluorescence polarization-based assay to disrupt FOXM1–DNA interaction [15]. A single-stranded DNA aptamer prevents the binding of FOXM1 DBD and inhibits FOXM1 transcriptional activities [16]. Based on the crystal structure of FoxM1-DBD in a complex with a tandem recognition sequence [17], we performed pharmacophore and structure-based approaches and successfully identified XST-20 that disrupts FOXM1-DBD and suppresses its transcriptional activities. More study is needed to improve the activity and solubility.

In summary, our study here identified a novel selective FOXM1 inhibitor using pharmacophore and structure-based approaches. Compound XST-20 directly interacts with FOXM1 DNA-binding domain determined by SPR assay. Furthermore, XST-20 was found to exhibit an anti-tumor effect on ovarian cancer cell lines. XST-20 significantly reduced the colony-forming efficiency and induced cell cycle arrest and apoptosis. Importantly, XST-20 greatly suppressed transcriptional activities of FOXM1. Our study provides a lead compound of FOXM1 inhibitor which might be as a potential targeted probe for ovarian cancer treatment.

## Materials and methods

### PyMOL for molecular visualization

The structure of the FOXM1–DNA complex (PDB ID: 3G73) was used to explore the interaction between FOXM1 and DNA. The PyMOL Molecular Graphics System was employed to analyze the hydrogen bonds formed between protein amino acids and DNA.

### Virtual screening

The SPECS database which contains 277,324 compounds was downloaded from the ZINC database which are commercially available for experimental screening. The 3D coordinates of compounds were generated and imported into the molecule module in DS 2.5 for subsequent structural filtration and virtual screening. SPECS database was filtered to exclude compounds that did not follow Lipinski and Veber rules.

Docking studies were performed using the Libdock module of DS package 2.5. For molecular docking, the 2.2 Å X-ray crystal structure FOXM1 (PDB ID: 3G73) was retrieved from the protein data bank (PDB). SYBYL-X 2.0 software was employed to calculate molecular docking. Firstly, the backbones and sidechain rotamers of FOXM1 were repaired. Next, the protein was prepared for molecular docking by removing crystal water and other hetero-atoms around it, followed by the addition of hydrogen, protonation, ionization, and assignment of atom type by Amber7-FF99 force field. The prepared protein was subsequently imported to Libdock. According to the previous research [17], the DNA binding site was defined near three residues as His-287, Arg-286, and Asn-283 and the regions within a radius of 9 Å. Then the CHARMm force field was conducted for ligands and receptor minimization. Libdock was used for structure-based virtual screening. After screening, all ligands were ranked and grouped based on the Libdock score.

The 400 top-scored molecules were selected to perform the ADMET prediction with the DS 2.5 software package by estimating the drug-likeness in silico. These studies were solely based on the chemical structure of the molecules. During the ADMET prediction, molecules were checked for non-CYP2D6 inhibition and non-hepatotoxicity. Other pharmacokinetic parameters included blood–brain barrier (BBB), solubility, and intestinal absorption, were evaluated by setting their values to 2, 2, and 0, respectively.

### Cell lines

Ovarian cancer cell lines A2780, HEY, and SKOV3 were obtained from Sigma-Aldrich (St. Louis, MO, USA). HO8910 was purchased from Shanghai Cell Bank, Chinese Academy of Sciences (Shanghai). A2780, HO8910, and HEY cells were cultured in Dulbecco’s modified Eagle’s medium (DMEM) (GIBCO, Waltham, MA, USA). SKOV3 cell line was cultured in RPMI 1640 medium (GIBCO, Waltham, MA, USA). All media contained 10% FBS, 100 μg/ml penicillin, and 100 μg/ml streptomycin.

### MTT assay

Ovarian cancer cells (3 × 10^3^/well) were seeded in 96-well plates and incubated overnight. The cells were then treated with various concentrations of compounds for 24, 48, or 72 h in triplicates. The MTT solution (5 mg/ml) was added to each well of the plate and the cells were incubated and the medium were carefully removed by aspiration. DMSO (100 μl) was then added to each well. The absorbance of each well was measured at 490 nm.

### Immunoblotting

Cells were harvested and lysed on ice in lysis buffer, the protein concentration was measured by BCA assay. About 30–50 μg protein samples were separated by SDS-PAGE (12%) and electro-transferred onto a PVDF membrane. The membrane was then blocked with 5% skim milk and incubated with specific primary antibodies at 4 °C overnight. Proteins of interest were detected with horseradish peroxidase (HRP)-conjugated secondary antibodies. β-actin or tubulin were used as loading controls. The primary antibodies were as follows: rabbit anti-p21Waf1/Cip1 (12D1) monoclonal antibody (8831, Cell Signaling Technology,1:1000); rabbit anti-CCNB1 polyclonal antibody (D160234,Sangon Biotech,1:500); mouse anti-cyclinD1(A-12) monoclonal antibody (sc-8396, Santa Cruz Biotechnology, 1:200); mouse anti-p27 Kip1 (F-8) monoclonal antibody (sc-1641, Santa Cruz Biotechnology, 1:200); mouse anti-Plk (F-8) monoclonal antibody (sc-17783, Santa Cruz Biotechnology,1:100); mouse anti-FOXM1(G-5) monoclonal antibody (sc-376471,Santa Cruz Biotechnology,1:100); mouse anti-β-actin monoclonal antibody (66009-l-lg,Proteintech, 1:8000); mouse anti-β-Tubulin (C66) monoclonal antibody (M20005S, Abmart, 1:5000).

### Surface plasmon resonance (SPR) assay

In this experiment, chemically synthesized FOXM1 (222–360) peptide (Purity (HPLC) >95%) was purchased from Genescript (Piscataway, NJ, USA). The SPR experiment were carried out on a Biacore T200 instrument (GE Healthcare). Briefly, FOXM1 protein was immobilized on a CM5 sensor chip via amine coupling reactions. The sensorgrams were obtained after sequential injection at different concentrations of of XST-20 (from 0.39 to 50 μM). All experiments were carried out at a constant temperature of 25.0 °C. Biacore T200 evaluation software was used with 1:1 binding model fitting for affinity evaluation (GE Healthcare).

### Luciferase reporter assay

PGL4 plasmids containing the CCNB1 promoter region were co-transfected with PCMV-FOXM1 and pRL-TK into HEK293T cells using lipofectamine 2000 (Invitrogen, USA). Luciferase activity was measured 48 h after transfection using the Dual-Luciferase Reporter Assay System (Promega). The relative luciferase activity was calculated as the ratio of firefly luminescence and Renilla luminescence.

### Clonogenic assay

Cells were seeded in six-well plates (1000–2000 cells per well) and incubated overnight. cells were then treated with different concentrations of the compound for 2 weeks. Colonies were fixed with methanol and stained with 0.1% crystal violet. Colonies of greater than 50 cells were counted. The data presented were represent three independent experiments.

### Apoptosis analysis

After being treated with different concentrations of XST-20 for 48 h, cells were harvested and stained with Alexa Fluor 488 annexin V and propidium iodide (Invitrogen) and analyzed with flow cytometry. Finally, the percentage of apoptotic cells were analyzed by FCS Express V3 software (Glendale, CA, USA).

### Statistical analysis

GraphPad Prism 8 was used for statistical analyses. Student’s *t*-test and one-way analysis of variance (ANOVA) were used to determine significant differences. The results are presented as the means ± SD of three independent experiments. Statistical significance was considered as *p < 0.05, **p < 0.01, ***p < 0.001, and ****p < 0.0001.

## Supplementary information

Supplementary Information file

Original Data File

## Data Availability

The data that support the findings of this study are available from the corresponding author upon reasonable request.
