# Supplementary material for: Small molecule targeting FOXM1 DNA binding domain exhibits anti-tumor activity in ovarian cancer
Source: Cell Death Discov. 2022 Jun 9;8:280. doi: 10.1038/s41420-022-01070-w (PMC9184618; doi:10.1038/s41420-022-01070-w)
Supplement: Supplementary file 1 — Supplementary Information file [file 41420_2022_1070_MOESM1_ESM.pdf]

Figure S1

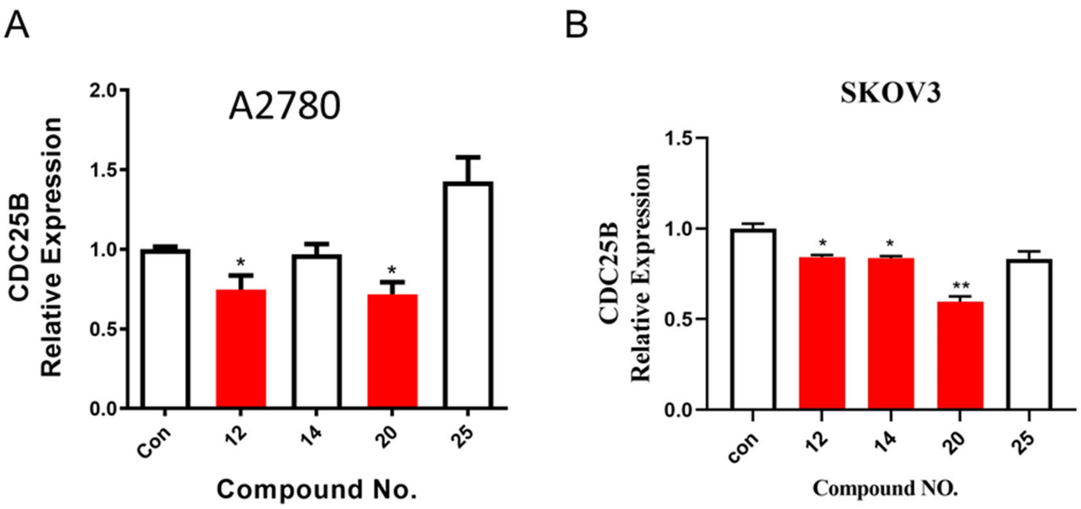

Expression level of CCNB1 was detected by qPCR in A2780 (A) and SKOV3 (B) cells treated with compounds for 48 hours.

Table S1. Compound number and docking scores of 15 candidates.

Compound	SPECS No.	Molweight	LibDock score
XST-1	AG-690/36788021	439.27	104.575
XST-2	AG-205/36483026	425.49	151.826
XST-4	AF-399/13375023	439.50	138.938
XST-6	AN-329/43449752	390.46	101.990
XST-7	AK-918/11755233	359.81	135.04
XST-11	AN-329/42138585	440.52	106.957
XST-12	AK-968/40709033	419.44	145.002
XST-14	AK-918/43446404	403.44	132.042
XST-20	AJ-292/42546280	355.48	131.129
XST-21	AP-970/42784485	435.55	100.162
XST-23	AQ-432/43399976	386.48	109.108
XST-24	AO-081/42097078	374.37	106.967
XST-25	AK-778/43420962	461.48	133.749
XST-26	AN-465/43030366	397.50	108.341
XST-27	AG-690/13116091	403.44	138.803
