# Supplementary material for: Small molecule targeting FOXM1 DNA binding domain exhibits anti-tumor activity in ovarian cancer
Source: Cell Death Discov. 2022 Jun 9;8:280. doi: 10.1038/s41420-022-01070-w (PMC9184618; doi:10.1038/s41420-022-01070-w)
Supplement: Supplementary file 2 — Original Data File [file 41420_2022_1070_MOESM2_ESM.docx]

Figuer4B

140kDa

140kDa

HEY

A2780


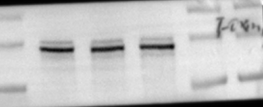

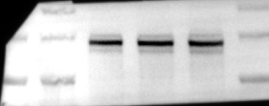


115kDa

80kDa

115kDa

80kDa

FOXM1


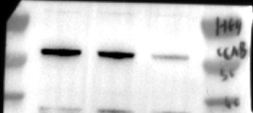

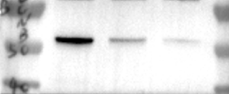


50kDa

65kDa

CCNB11

65kDa

50kDa


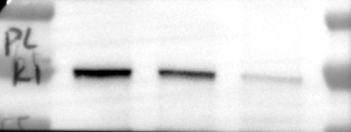

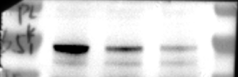


80kDa

40kDa

80kDa

40kDa

PLK1


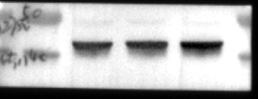


50kDa

65kDa

65kDa

50kDa


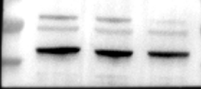


40kDa

40kDa

Actin


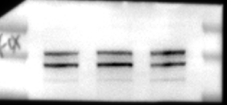


140kDa

115kDa

SKOV3

FOXM1


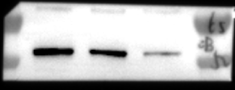


65kDa

80kDa

50kDa

CCNB11


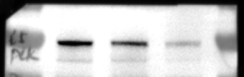


65kDa

80kDa

PLK1


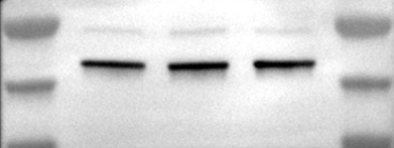


40kDa

50kDa

Actin


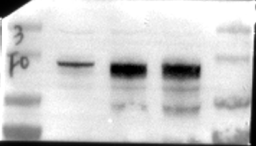


Figuer4C

115kDa

140kDa

80kDa


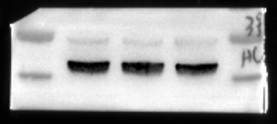


40kDa

50kDa

**Firure5C**

SKOV3

HEY

**A2780**


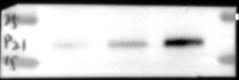

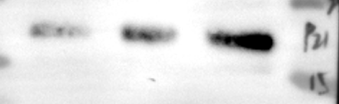

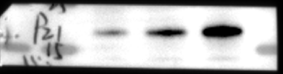


25kDa

15kDa

P21


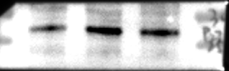

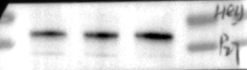

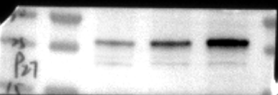


30kDa

P27


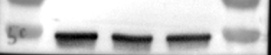

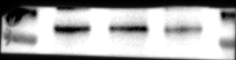

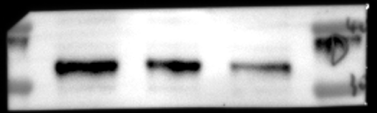

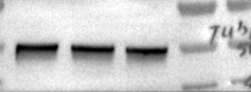

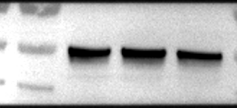

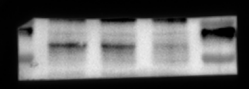


50kDa

30kDa

40kDa

25kDa

Cyclind

Tubulin
